# Association of *ACE1* I/D polymorphism and susceptibility to COVID-19 in Egyptian children and adolescents

**DOI:** 10.1038/s41390-023-02982-8

**Published:** 2024-01-04

**Authors:** Naglaa F. Boraey, Marwa A. Bebars, Ali A. Wahba, Hanan M. Abd El Lateef, Mohamed Atif Attia, Ahmed H. Elsayed, Khalid A. Rashed, Ehab I. Sorour, Mohamed F. Ahmed, Ghada A. B. Abd-Elrehim, Attia A. Soliman, Mohamed M. M. Shehab, Eman M. Elhindawy, Ahmed A. A. Ibraheem, Hassan Shehata, Yousif M. Yousif, Mustafa I. A. Hashem, Amani A. Ahmed, Ahmed A. Emam, Dalia M. Gameil, Eman M. Abdelhady, Khalil Abdelkhalek, Walaa E. M. A. Morsi, Dalia M. Selim, Suzan A. Razek, Bassem Ashraf, Ahmed S. E. Saleh, Heba H. Eltrawy, Mohamed I. Alanwar, Rania A. Fouad, Walaa E. Omar, Rehab M. Nabil, Mohamed R. Abdelhamed, Mona Yousri Ibrahim, Mai M. Malek, Mona R. Afify, Mohanned T. Alharbi, Mohammed K. Nagshabandi, Muyassar K. Tarabulsi, Mohammed Esmail Qashqary, Laila M. Almoraie, Hanan F. Salem, Manal M. Rashad, Sonya A. A. El-Gaaly, Nahawand A. El- Deeb, Amany M. Abdallah, Ahmed R. Fakhreldin, Mohamed Hassouba, Yasmine M. Massoud, Mona S. M. Attaya, Mohammed K. Haridi

**Affiliations:** 1 Department of Pediatrics, Faculty of Medicine, Sohag University, Sohag, Egypt; 2 Department of Pediatrics, Princess Alexandra hospital, Harlow, UK; 3 Department of Pediatrics at SSMC (Sheikh Shakhbout Medical City, Abu Dhabi, UAE; 4 Department of Pediatrics, Faculty of Medicine, Ain-Shams University, Cairo, Egypt; 5 Department of Pediatrics at SKMC (Sheikh khalifa Medical City, Abu Dhabi, UAE; 6 Department of Pediatrics, Faculty of Medicine for Boys, Al-Azhar University, Al-Azhar, Egypt; 7 Department of Pediatrics, Faculty of Medicine, Zagazig University, Zagazig, Egypt; 8 Department of Pediatrics, Faculty of Medicine, Cairo University, Cairo, Egypt; 9 Department of Otorhinolaryngology, Faculty of Medicine, Mansoura University, Mansoura, Egypt; 10 Department of Otorhinolaryngology, Faculty of Medicine, Benha University, Benha, Egypt; 11 Department of Chest diseases, Faculty of Medicine for Girls, Al-Azhar University, Al-Azhar, Egypt; 12 Department of Cardiothoracic surgery, Faculty of Medicine, Zagazig University, Zagazig, Egypt; 13 Department of Medical Biochemistry, College of Medicine, AlMaarefa University, Riyadh, Saudi Arabia; 14 Department of Medical Biochemistry, Faculty of Medicine, Zagazig University, Zagazig, Egypt; 15 Department of Clinical Pathology, Faculty of Medicine, Zagazig University, Zagazig, Egypt; 16 Department of Clinical pathology, Faculty of Medicine for Boys, Al-Azhar University, Al-Azhar, Egypt; 17 Department of Clinical pathology, Faculty of Medicine for Girls, Al-Azhar University, Al-Azhar, Egypt; 18 Department of Microbiology and Immunology, Faculty of Medicine, Zagazig University, Zagazig, Egypt; 19 Department of Medical microbiology and Parasitology. Faculty of Medicine, University of Jeddah, Jeddah, 21589 Saudi Arabia; 20 Department of Family and community medicine, University Medical Center, University of Jeddah, Jeddah, Saudi Arabia; 21 Department of Anesthesia, Faculty of Medicine, Benha University, Banha, Egypt; 22 Department of Anesthesia, Faculty of Medicine, Zagazig University, Zagazig, Egypt; 23 Department of Internal Medicine, Faculty of Medicine, Ain-Shams University, Ain-Shams, Egypt; 24 Department of Internal Medicine, Faculty of Medicine, Zagazig University, Zagazig, Egypt; 25 Department of Family Medicine, Faculty of Medicine, Zagazig University, Zagazig, Egypt; 26 Department of Pediatrics, Faculty of Medicine, Aswan University, Aswan, Egypt; 27 Department of Pediatrics, SUNY Downstate Health Science University, Kings County Hospital, Brooklyn, NY USA; 28 Department of Tropical Medicine, Faculty of Medicine, Ain-Shams University, Ain-Shams, Egypt; 29 Department of Pediatrics, Faculty of Medicine for Girls, Al-Azhar University, Al-Azhar, Egypt; 30 Department of Pediatrics, Faculty of Medicine, Assiut University, Assiut, Egypt

## Abstract

**Background:**

Given the sparse data on the renin-angiotensin system (RAS) and its biological effector molecules ACE1 and ACE2 in pediatric COVID-19 cases, we investigated whether the *ACE1* insertion/deletion (I/D) polymorphism could be a genetic marker for susceptibility to COVID-19 in Egyptian children and adolescents.

**Methods:**

This was a case-control study included four hundred sixty patients diagnosed with COVID-19, and 460 well-matched healthy control children and adolescents. The I/D polymorphism (rs1799752) in the *ACE1* gene was genotyped by polymerase chain reaction (*PCR*), meanwhile the ACE serum concentrations were assessed by *ELISA*.

**Results:**

The *ACE1* D/D genotype and Deletion allele were significantly more represented in patients with COVID-19 compared to the control group (55% vs. 28%; OR = 2.4; [95% CI: 1.46–3.95]; for the DD genotype; *P* = 0.002) and (68% vs. 52.5%; OR: 1.93; [95% CI: 1.49–2.5] for the D allele; *P* = 0.032). The presence of *ACE1* D/D genotype was an independent risk factor for severe COVID-19 among studied patients (adjusted OR: 2.6; [95% CI: 1.6–9.7]; *P* < 0.001.

**Conclusions:**

The *ACE1* insertion/deletion polymorphism may confer susceptibility to SARS-CoV-2 infection in Egyptian children and adolescents.

**Impact:**

- Recent studies suggested a crucial role of renin-angiotensin system and its biological effector molecules ACE1 and ACE2 in the pathogenesis and progression of COVID-19.
- To our knowledge, ours is the first study to investigate the association of ACE1 I/D polymorphism and susceptibility to COVID-19 in Caucasian children and adolescents.
- The presence of the ACE1 D/D genotype or ACE1 Deletion allele may confer susceptibility to SARS-CoV-2 infection and being associated with higher ACE serum levels; may constitute independent risk factors for severe COVID-19.
- The ACE1 I/D genotyping help design further clinical trials reconsidering RAS-pathway antagonists to achieve more efficient targeted therapies.

## Introduction

Coronavirus disease 2019 (COVID-19) is a global pandemic caused by an enveloped, single-stranded RNA -β coronavirus that was later named severe acute respiratory syndrome coronavirus 2 (SARS-CoV-2).^1^ Since January 2020, more than 600 million confirmed cases and 6 million deaths have been reported worldwide.^2^

COVID-19 has been widely reported to be less severe in children and young people with mostly asymptomatic or mild cases and reports of death are scarce.^3^ However, a more complicated course has been described in a subset of pediatric patients with the development of severe pneumonia, acute respiratory distress syndrome (ARDS), septic shock, coagulopathy and multi-organ failure.^4^

The SARS-CoV-2 spike glycoprotein recognizes and binds to human angiotensin-converting enzyme 2 (ACE2), the functional receptor for SARS-CoV-2 to enter the target cells.^5^ ACE2 is mainly expressed on type II alveolar cells, myocardial cells, proximal renal tubular cells as well as the enterocytes of the small intestine.^6^

The angiotensin I-converting enzyme1 (ACE1) and its homologue ACE2 are key biomolecules for the tuning of the renin–angiotensin system (RAS); the homeostatic regulator of vascular function with prominent effect on the renal, vascular, cardiac, and immune system.^7^ ACE1 converts angiotensin I into vasoactive angiotensin II, stimulating vasoconstriction and promoting inflammatory and thrombotic process as well as alveolar epithelial cell apoptosis through binding to angiotensin type 1 (AT1) receptors. In sharp contrast, ACE2 converts angiotensin II into Angiotensin 1–7 which binds to Mas receptors to counteract the negative effects of Angiotensin II.^8^ Several studies reported that down-regulation of ACE2 expression and increased Angiotensin II production during SARS-CoV2 infection resulted in severe lung injury, suggesting a crucial role of RAS and associated signaling cascade in the pathogenesis and progression of COVID-19.^9,10^

In animal models of acute lung injury, Angiotensin II is up-regulated by enhanced ACE activity resulting in increased vascular permeability, massive pulmonary edema, impaired oxygenation and rapid worsening of lung function. Injection of recombinant h-ACE2 protein was protective against severe acute lung injury in these models.^11^

In humans, the *ACE1* gene is located on chromosome 17 (17q23.3) and consists of 25 introns and 26 exons. Among several polymorphisms, the *ACE1 I/D* gene polymorphism refer to the insertion (*I*) or deletion (*D*) of a 287 base-pair sequence within intron 16.^12^ This functional polymorphism accounts for about 47% of the phenotypic variations in circulating and tissue ACE activity. Subjects carrying the *ACE1* D allele were found to have approximately two-fold increased systemic ACE level, whereas those with the *ACE1* II genotype have the lowest ACE expression.^13^ Previous studies reported that the *ACE1* deletion allele is associated with increased risk and severity of community acquired pneumonia (CAP), acute respiratory distress syndrome (ARDS) and meningococcal disease in Caucasian children.^14–16^

Given the sparse data on the renin-angiotensin system (RAS) and its biological effector molecules ACE1 and ACE2 in pediatric COVID-19 cases, we investigated whether the *ACE1* I/D polymorphism (rs1799752) could be a genetic marker for susceptibility to COVID-19 in Egyptian children and adolescents.

## Methods

This was a prospective multicenter study conducted at Zagazig, Cairo and Ain-Shams University hospitals from January 2020 through June 2021.

### Ethics statement

The study protocol was approved by the medical Ethics Committees at Zagazig, Cairo and Ain-Shams Universities, Egypt. Written informed consent was provided by parents or legal guardian for each participant. The study was performed in accordance with the Declaration of Helsinki.

### Case definition

Four hundred and sixty unrelated patients, who were diagnosed to have COVID‐19 on hospital admission, were included. Patients aged less than 19 years and tested positive for SARS-CoV-2 by real-time reverse-transcriptase (RT)-PCR assay were accepted as laboratory confirmed cases. Pulmonary high-resolution CT images were routinely performed and evaluated for all participants. The severity of COVID-19 was further classified into five subgroups (asymptomatic, mild, moderate, severe, and critical) according to the clinical presentation combined with chest radiograph imaging and laboratory testing.^17^

- Critical subgroup: patients who required intensive care unit monitoring for acute respiratory failure, shock, or organ failure.

Acute respiratory failure was defined according to Berlin’s criteria.^18^ Shock was defined according to the Pediatric Sepsis Consensus Conference.^19^

- Severe subgroup: cases who rapidly developed hypoxia, dyspnea, dehydration, gastrointestinal dysfunction, encephalopathy, coagulopathy, acute renal injury or other vital organs injury.
- Moderate subgroup: cases having clinical signs of pneumonia plus fever >38 °C and age-specific tachypnea but not fulfilling criteria of severe pneumonia as classified by ref. ^20^
- Mild subgroup: patients tested positive for SARS-CoV-2 by RT‐PCR showing upper respiratory symptoms but no signs of viral pneumonia or abnormal radiographic findings.
- No asymptomatic COVID-19 cases were seen among studied cohort.

### Exclusion criteria

Patients with malnutrition; heart disease; malignancy; autoimmune disorders; primary or acquired immunodeficiency; or any chronic or metabolic disease were excluded. Patients hospitalized within the past 30 days or those on immunosuppressive drugs or *ACE* inhibitors and obese subjects were also excluded.

### Control group

Four hundred and sixty unrelated healthy children and adolescents, of matched age and sex, were recruited as a control group during their routine medical checkup at the outpatient clinics for children and adolescents in the study hospitals (all had negative anti-N antibodies test for SARS-Cov2 and tested negative by RT-PCR and without a previous history or diagnosis of LRI).

Both patient and control groups belong to the same ethnicity: African Caucasian.

Upon enrollment, demographic information, medical history, clinical data and laboratory results were recorded for all subjects. Five ml whole venous blood samples were withdrawn for serological and molecular analysis. Routine laboratory investigations included CBC, C‐reactive protein (CRP), Procalcitonin, D-Dimer, serum ferritin, and Lactate dehydrogenase (LDH).

### SARS- CoV-2 diagnosis

For all participants, nasal or pharyngeal swab, were obtained and transferred immediately to the medical molecular laboratory of the study hospitals. The presence of SARS-CoV-2 was detected by real-time transcription PCR using Bio-Speedy® SARS-CoV-2 Triple Gene RT-qPCR kit (Bioeksen, İstanbul, TÜRKIYE) according to the manufacturer protocol. This kit is a real-time and one-step reverse transcription PCR test targets the *Orf1ab, N, and E* genes in the SARS-CoV-2 genome with sensitivity: 100% and specificity**:** 100%.

The SARS-CoV-2-positive specimens were also tested for other respiratory pathogens using the multiplex RT-PCR Diagnostics Resp21 panel (Fast-Track Diagnostics, Luxembourg).

### Estimation of serum ACE concentrations

Serum ACE concentrations were estimated by ELISA (Human ACE/Cd143 ELISA Kit PicoKine® (BosterBio Tech., Pleasanton CA, Catalog # EK0557) according to the manufacturer’s instructions with Sensitivity: <5 pg/ml.

### Genotyping of the *ACE* gene I/D polymorphism

Genomic DNA was extracted from 200 μL of frozen anti-coagulated blood using the QIAamp Blood kit (Qiagen, Germany) and then stored at −20 °C until genotyping.

All participants were genotyped for the *ACE1* gene I/D polymorphism ((rs1799752) by polymerase chain reaction technique (PCR) as previously described.^21^

The sequences of PCR primers were: the sense primer 5′-CTG GAG ACC ACT CCC ATC CTT TCT 3′, and the antisense primer 5′-GAT GTG GCC ATC ACA TTC GTC AGA T 3′. The initial denaturation step at 94 °C for 5 min was followed by annealing 30 cycles at 58 °C for 30 s; and a final extension at 72 °C for 5 min. PCR products were visualized on 3% agarose gel mixed with ethidium bromide and viewed under UV illuminator. This PCR method resulted in a 190-bp product (D allele) and a 490-bp product (I allele). Two bands of 490 and 190 bp were detected in heterozygote samples.

### Statistical analysis

Statistical analysis was performed using SPSS (IBM statistics, Chicago) version 22 and the Open Epi Info software package version 2.3.1 *(*www.openepi.com). The Chi-square (χ^2^) test was applied to compare categorical variables and to evaluate the Hardy–Weinberg equilibrium (HWE) for the distribution of the *ACE1* I/D genotypes of the patients and the controls. Continuous parameters were compared by Student’s *t*‐test, or analysis of variance (ANOVA)-test, as appropriate. Logistic regression analysis was applied to explore independent variables that were associated with disease severity. Odd ratio with 95% confidence interval [OR; 95% CI] were computed. A *P* < 0.05 was considered as significant.

## Results

Over the 18-months study period, 460 patients with laboratory confirmed COVID-19 and 460 healthy control subjects were enrolled. Patients had a median age of 9.5 years; range (8–19) years and 299 (65%) were males. The control group were well-matched for age, and sex [median age 9.8 years; range (8–18.9) years] and 290 (63%) were males; (*P* = 0.563, *P* = 0.217, respectively); Table 1. A definitive history of contact with a family member with COVID-19 was reported in (93%) of patients. Fever, dry cough and dyspnea were the most prevalent clinical presentations on admission. Other symptoms were infrequently recorded as listed in Table 1.

**Table 1:** The demographic data, clinical features and laboratory results of COVID-19 patients.

	Patients (*n* = 460)	Control group (*n* = 460)	*P*
Age (years)	9.5 (8–19)	9.8 (8–18.9)	0.563
Male, *n* (%)	299 (65)	290 (63)	0.217
Family member with COVID-19	428 (93)	_	_
Risk factors		_	_
History of asthma	78 (17)		
Passive smoking	64 (14)		
Signs or symptoms of LRTI	198 (43)		
COVID-19 Severity			
Mild	147 (32)		
Moderate	166 (36)		
Severe	87 (19)		
Critical	60 (13)		
Fever >38 °C	460 (100)		
Dry cough	258 (56)	_	_
Dyspnea	120 (26)	_	_
Nausea/vomiting	106 (23)		
Diarrhea	87 (19)	_	_
Dehydration	55 (12)		
Sore throat	64 (14)	_	_
Loss of taste	51 (11)	_	_
Anosmia	41 (9)	_	_
Fatigue	60 (13)	_	_
Hypoxemia	69 (15)	_	_
Cyanosis	46 (10)	_	_
Pneumonia	198 (43)		
ICU admission	60 (13)		
Shock	32 (7)	_	_
ARF	28 (6)		
Duration of fever (in days)	4 (3–9)		
Duration of hospital stay (in days)	9 (7–21)	_	_
Some risk factors			
History of asthma	37 (8)	26 (5.6)	0.316
Passive smoking	64 (14)	51 (11)	0.642
Signs or symptoms of LRTI	198 (43)	_	_
Viral co-infection			
Influenza B	23 (5)	_	
Picornaviruses	18 (4)		
HCoV-NL63	9 (2)	_	
h-MPV	5 (1)		
Chest CT Scan		_	_
Ground-glass opacities	198 (43)		
Pulmonary consolidation	189 (41)		
Laboratory findings, median (IQR)			
CRP, mg/dl	7.6 (0.98–25)		
Increased, *n* (%)	179 (39)		
Procalcitonin, ng/ml	14 (1.6–18.9)		
Increased, *n* (%)	212 (46)		
Serum Ferritin, ng/ml	43 (29–385)		
Lactate dehydrogenase (LDH), U/L	240 (198–307)		
D-Dimer, μg/mL	0.68 (0.37–3.5)		
Increased, *n* (%)	244 (53)		
White blood cell, ×10 ^9^/L	7.6 (5.9–11.7)		
Leucopenia, *n* (%)	78 (17)		
Lymphocytes, ×10^9^/L	2.34 (1.2–3.1)		
Lymphopenia, *n* (%)	152 (33)		
Platelets, ×10^9^/L	169 (138–319)		
Thrombocytopenia, *n* (%)	97 (21)		
ALT, U/L	21 (17–86)		
Increased, *n* (%)	78 (17)		
AST, U/L	28 (19–96)		
Increased, *n* (%)	110 (24)		
Creatinine, μmol/L	43 (28–79)		
Increased, *n* (%)	87 (19)		

Data are median (IQR) or *n* (%).

Reference values; CRP, <8 mg/dl; Procalcitonin, <0.5 ng/ml; Serum Ferritin, 15–140 ng/ml; D-Dimer, <0.5 μg/mL; LDH, 155–235 U/L; ALT, 9–50 U/L; AST, 5–60 U/L; Serum Creatinine, 18–62 μmol/L.

*ARF* acute respiratory failure, *IQR* interquartile range, *CRP* C‐reactive protein, *ALT* alanine aminotransferase, *AST* aspartate aminotransferase, *CT* computed tomography, *HCoV* human coronavirus, *h-MPV* human metapneumovirus.

Among studied cohort, 198 patients (43%) had pneumonia. According to disease severity, 147 (32%) patients had mild illness; 166 (36%) had moderate clinical type; 87 (19%) cases had severe COVID-19 and 60 (13%) were critical cases. Sixty cases admitted to ICU; 32 patients (7%) had shock and 28 (6%) cases required mechanical ventilation for ARF. No asymptomatic cases were seen among our cohort and all patients survived. Abnormal laboratory findings were decreased lymphocytes (152 [33%]), leucopenia (78 [17%]), and elevated D-dimer level (53%; median: 0.68 μg/mL [IQR 0.37–3.5]); Table 1. The baseline demographic, clinical data, radiological and laboratory findings of patients and control group are presented in Table 1. Fifty five patients (12%) were co-infected with at least one additional respiratory virus. The identified viruses were influenza B (5%), picornaviruses (4%), human coronavirus (HCoV-NL63) (2%) and human metapneumovirus (hMPV) (1%). Neither influenza A nor respiratory syncytial virus were detected; Table 1. We compared clinical and laboratory variables of COVID-19 patients with varying degrees of severity. Of note, lymphocyte count and D-Dimer level were significantly different among studied subgroups (*P* = 0.006, *P* = 0.02, respectively); Supplementary Table S1.

The *ACE1* I/D genotype distribution in patients with COVID-19 and control group were compatible with the Hardy–Weinberg equilibrium and are summarized in Table 2. The *ACE1* D/D genotype was more represented in patients with COVID-19 compared to the control group (55% vs. 28%, respectively). The *ACE1* D/D homozygous patients had 2.4-fold increased susceptibility to COVID-19 (OR = 2.4; [95% CI: 1.46–3.95]; *P* = 0.002); Table 2. Besides, *ACE1* D allele was significantly more frequent among studied patients compared to the control group (68% vs. 52.5%; OR: 1.93; [95% CI: 1.49–2.5]; *P* = 0.032); Table 2. Of note, patients with COVID-19 had significantly higher serum ACE concentrations as compared to the control group (mean; 68.7 ± 11.5 pg/ml vs. 23.5 ± 9.8 pg/ml respectively; *P* < 0.01); Table 2.

**Table 2:** Distribution of the angiotensin-converting enzyme1 (*ACE1*) I/D genotypes, allele frequency and serum ACE in patients with COVID-19 and control group.

		Patient group	Control group	OR (95% CI)	*P*
		*n* (460) %	*n* (460) %		
*ACE1* I/D Genotype	I/I	87 (19)	106 (23)	Referent	
	I/D	120 (26)	225 (49)	0.66 (0.39–1.09)	0.083
	D/D	253 (55)	129 (28)	2.4 (1.46–3.95)	0.002
Alleles	I	294 (32)	437 (47.5)	0.52 (0.40–0.67)	
	D	626 (68)	483 (52.5)	1.93 (1.49–2.5)	0.032
serum ACE (pg/mL)		68.7 ± 11.5	23.5 ± 9.8		< 0.01^a^

Values in parentheses are percentages or data are presented as mean ± SD.

*COVID-19* coronavirus disease 2019, *ACE* angiotensin-converting enzyme, *OR* odds ratio, *CI* 95% confidence interval.

*P* < 0.05 indicates a significant difference. Chi-square test.

^a^Student *t*-test.

As regards COVID-19 severity, the homozygous *ACE1* D/D genotype was more frequent among severe and critical cases 62 (24.5%) and 41 (16%); respectively, *P* < 0.001; Table 3. In addition, patients carrying the *ACE1* D/D genotype were more likely to have acute respiratory failure compared to those with the *ACE1* I/D or I/I genotypes (OR: 3.8; [95% CI: 1.03–12.15]; *P* = 0.02). No significant association was evident between the *ACE1* I/D genotypes and the risk of shock among studied patients; *P* = 0.324; Table 3. The *ACE1* D/D genotype was also more frequent in patients admitted to ICU due to COVID-19 (16%) compared to those with the *ACE1* I/D or I/I genotypes [(10%) and (8%); respectively, *P* < 0.01]; Table 3.

**Table 3:** Association of the *ACE1* I/D genotypes with COVID-19 severity, and clinical outcome among studied patients.

*ACE1* I/D Genotype	I/I (*n* = 87)	I/D (*n* = 120)	D/D (*n* = 253)	*P*
	*n* (%)	*n* (%)	*n* (%)	
COVID-19 severity				
Mild	57 (65)^a^	45 (38)	45 (18)	
Moderate	14 (16)	47 (39)	105 (41.5)	
Severe	9 (10)	16 (13)	62 (24.5)^a^	0.001
Critical	7 (8)	12 (10)	41 (16)^a^	0.001
Shock (*n* = 32)	5 (6)	8 (7)	19 (7.5)	0.324
ARF (*n* = 28)	2 (2)	4 (3)	22 (9)^a^	0.01
Clinical outcome				
ICU admission (*n* = 60)	7 (8)	12 (10)	41 (16)^a^	0.01
In-Hospital mortality	0 (0.0)	0 (0.0)	0 (0.0)	NA

*COVID-19* coronavirus disease 2019, *ACE* angiotensin-converting enzyme, *ARF* Acute respiratory failure, *ICU* intensive care unit.

*P* < 0.05 indicates a significant difference.

^a^Significant difference between each three genotypes group.

**Fig. 1:**
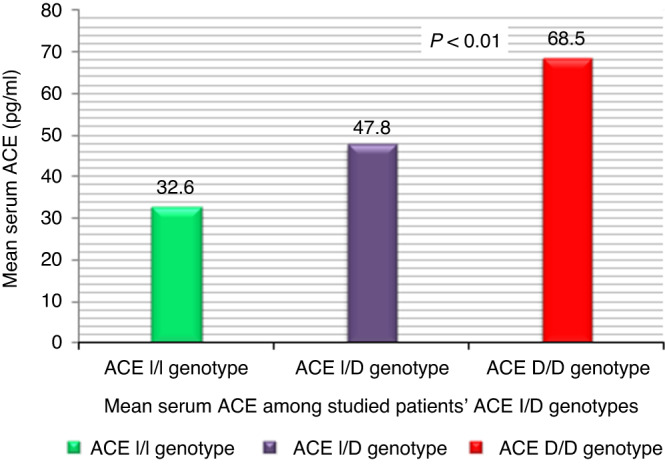
Mean serum Angiotensin Converting Enzyme (ACE) among studied patients’ *ACE1* I/D genotypes.

Patients with *ACE1*D/D genotype had significantly higher mean ACE serum level compared to those with I/D and I/I genotypes (68.5 ± 12.6 pg/ml vs. 47.8 ± 9.7 and 32.6 ± 11 pg/ml, respectively); *P* = 0.015, Table 4; Fig. 1.

**Table 4:** Comparison of ACE serum levels in patients with COVID-19 with various *ACE1 I/D* genotypes, disease severity and adverse outcome.

Characteristics	Serum ACE (pg/mL)	*P*	
*ACE1* Genotype			
I/I	32.6 ± 11		
I/D	47.8 ± 9.7		
D/D	68.5 ± 12.6^a^		0.015*
Disease severity			
Mild	31.5 ± 8.6		
Moderate	43.6 ± 10.5		
Severe + critical	67.8 ± 13^a^		0.032
Non-ICU patients	34.6 ± 9.5		
ICU patients	65.7 ± 12.8		0.008

Data are presented as mean ± SD.

*COVID-19* coronavirus disease 2019, *ACE* angiotensin-converting enzyme, *ICU* intensive care unit.

*P* < 0.05 indicates a significant difference.

*ANOVA test.

^a^Significant difference between each three genotypes group.

In addition, severe and critical cases had significantly higher ACE serum levels (mean, 67.8 ± 13 pg/ml) compared to those with mild or moderate COVID-19 (31.5 ± 8.6 pg/ml and 43.6 ± 10.5 pg/ml; respectively); *P* = 0.03, Table 4. Mean ACE serum level was significantly higher in patients who required ICU supportive care than non-ICU patients (65.7 ± 12.8 vs. 34.6 ± 9.5 pg/ml); *P* = 0.008, Table 4.

Serum ACE level was negatively correlated with lymphocyte count and oxygen saturation among studied patients (*r* = − 0.374, *P* = 0.04; and *r* = − 0.365, *P* = 0.02, respectively); meanwhile no significant correlations were found between ACE serum levels and other measured laboratory or clinical parameters (Supplementary Table S2).

We further categorized patients with severe and critical illness as severe COVID-19 who was compared to non-severe COVID-19 which included mild and moderate clinical subtypes. Logistic regression model showed that the presence of *ACE1* D/D genotype or D allele was an independent risk factor for severe COVID-19 among studied patients (adjusted OR: 2.6; [95% CI: 1.6–9.7]; *P* < 0.001, for the *ACE1* D/D genotype and adjusted OR: 1.96; [95% CI: 1.4–3.8]; *P* < 0.01 for the *ACE1* D allele) controlling for age, sex and viral co-infection; Table 5.

**Table 5:** Risk factors for severe COVID-19 among studied patients.

Covariate	Univariate analysi		Multivariate analysis	
	Unadjusted OR (95%CI)	*P*	^a^adjusted OR (95%CI)	*p*
Age	0.8 (0·4–1·6)	0.20		
Male	1.2 (0·9–3·8)	0.13		
Weight for age z-scores	0.93 (0.58–1.3)	0.57		
History of asthma	1.6 (0.7–14)	0.26		
Passive smoking	2.34 (0.93–1.78)	0.74		
Signs or symptoms of LRTI	0.94 (0.6–1.46)	0.86		
Viral co-infection	1.5 (0.83–6.2)	0.18		
Bacteremia	1.3 (0.73–1.9)	0.67		
*ACE1 I/D* Genotype				
(D/D vs I/D + I/I)	2.4 (1.46–3.95)	0.002	2.6 (1.6–9.7)	< 0.001
ACE Allele (D vs I)	1.9 (1.49–2.5)	0.032	1.96 (1.4–3.8)	< 0.01

*COVID-19* coronavirus disease 2019, *ACE* angiotensin-converting enzyme, *LRTI* lower respiratory tract infection.

*P* < 0.05 indicates a significant difference.

^a^Adjusted for age, sex and viral co-infection.

## Discussion

The *ACE1* I/D SNP has been linked to a variety of renal, cardiovascular and inflammatory human diseases.^22,23^ The *ACE1* deletion allele has been associated with increased risk and severity of community acquired pneumonia, acute respiratory distress syndrome and sepsis in Caucasian children.^14,15,24^

Delanghe et al. reported that the prevalence of COVID-19 was strongly correlated with the *ACE1* I/D polymorphism in European, North-African and Middle East countries and may be regarded as a biomarker for historical migration of humans with causal genetic factors involved in the spread of SARS-CoV-2 infection.^25^

To the best of our knowledge, ours is the first study to investigate the *ACE1I/D* polymorphism in Caucasian children and adolescents with COVID-19.

Consistent with a recent meta-analysis by de Souza et al.^26^ fever, dry cough and shortness of breath were the most prevalent symptoms among studied cohort followed by vomiting and diarrhea. The most common abnormal laboratory findings were reduced lymphocyte counts and elevated D-dimer level and these parameters were significantly associated with disease severity among studied patients.

In this study, the homozygous *ACE1* D/D genotype and D allele were significantly more represented in patients with COVID-19 compared to the control group. In addition, patients carrying the *ACE1* D/D genotype had 2.4- fold increased susceptibility to SARS-CoV-2 infection. By contrast, the *ACE1 I/I* genotype and

*I* allele were less frequent in patients with COVID-19 than controls suggesting that the *ACE I* allele may confer protection against COVID-19. Of note, severe and critical cases exhibited significantly higher frequency of the *ACE1* D/D genotype than those with mild or moderate COVID-19.

In accordance with our results, recent studies reported that the D allele of *ACE1* and DD genotype is integrally involved in susceptibility to SARS-CoV-2 infection and COVID-19 progression, meanwhile the *ACE1*-II genotype negatively correlates with infection rate and hospital mortality.^27,28^ Verma et al. reported that carrying the *ACE1* D/D genotype was associated with a 3.6-fold higher risk of severe COVID-19 in Indian population.^29^

ACE1 and ACE2 counteract each other in the RAS cascade to balance the ACE/Angiotensin II/AT1R axis, which is a pro-inflammatory, and vasoconstrictor pathway along with the ACE2/ Angiotensin 1–7/Mas receptor signaling pathway that promotes anti-inflammatory and vasodilator effects. This balance protects against vascular pathology and organ dysfunction by anti-inflammatory, anticoagulant, anti-alveolar epithelial cell apoptosis, anti-fibrotic and anti-oxidative stress effects antagonizing the Angiotensin-II effects.^7,8^

Since the ACE1/ACE2 balance is crucial to contrast organ dysfunction, polymorphisms in the *ACE1* gene that affect its expression level, may accelerate lung damage and pulmonary shut-down triggered by SARS-CoV-2 infection.^10,11,30^

In an attempt to explain our findings, we have investigated ACE serum level in patients with COVID-19 which was significantly higher compared to the control group. There is conflicting data as regards to ACE levels in COVID-19 cases. Some studies revealed an increased ACE serum levels,^31^ while others reported similar systemic ACE activity in patients with COVID-19 and healthy adults.^32^ However, data regarding ACE levels in children and adolescents infected with SARS-CoV-2 is still lacking.

Moreover, we found that patients homozygous for the *ACE1* D/D genotype had significantly higher systemic ACE levels, being approximately twice as compared to those carrying I/I genotype, whereas those with I/D genotype have intermediate concentrations. The *ACE1* I/D polymorphism accounts for approximately half (47%) variation in ACE mRNA expression and activity.^13^ To date, only a few studies in pediatric population investigated the possible role of *ACE1* rs1799752 alleles and ACE serum levels.^22,33^ Together with our findings, it confirm and extend earlier studies in adult subjects that *ACE1* D-allele carriers show higher serum ACE levels than carriers of the *ACE1* I- allele.^13,25,34–36^

Of note, serum ACE level was constantly increased across the severity of COVID-19 as severe and critical cases had markedly elevated ACE serum levels compared to those with moderate and mild clinical types. In addition, patients who required ICU supportive care had significantly higher ACE serum levels than non-ICU patients. Serum ACE level was negatively correlated with lymphocyte count and oxygen saturation among studied patients, meanwhile no significant correlations were found between ACE serum levels and other measured inflammatory biomarkers or clinical parameters.

Therefore, the current study demonstrates that the presence of the *ACE1* D/D genotype; being associated with higher ACE serum levels; may constitute risk factors for more severe COVID-19 clinical course as well as developing ARF, and ICU admission. However, no significant association was evident between the *ACE1* I/D genotypes and the risk of shock among studied patients.

Our findings also resonate with those of Abouzeid et al. who studied the *ACE1* I/D polymorphism on genomic DNA of 300 children diagnosed with CAP. The authors reported that the *ACE1* deletion allele and D/D genotype were associated with higher ACE activity and increased risk for severity and adverse outcome of CAP in Egyptian children.^14^ Similar study reported a higher frequency of the *ACE1* D allele in Vietnamese SARS cases who require supplementary oxygen than in the non-hypoxemic group. The authors suggested that the *ACE1* gene may be a candidate gene that influences the progression of pneumonia in SARS.^36^ On the contrary, Gómez et al. reported that the *ACE1* I/D SNP has no effect on the risk for COVID-19 in Spanish population although it may influence disease severity.^37^ Avanoglu Guler et al. reported that serum ACE activity did not correlate with inflammatory biomarkers and has not been related to disease severity or clinical outcome in Turkish population.^32^ Ethnic variations and differences in cohort size and/or studied age group as well as gene-environmental interplay may contribute to this discrepancy among different populations. A worldwide geographic distribution showed a decline of the *ACE1* D-allele from the highest frequencies in African American, Europe, and Arab region (from 0.57 up to 0.88), towards an intermediate frequency in Australia and America with the lowest frequency locates in Eastern Asian population (0.12 to 0.27).^38^ However, only two studies reported the *ACE1* I/D rs1799752 genotype distribution in children and adolescent age group. Ajala et al. reported that (0.39) were homozygous for the *ACE1* D allele, (0.14) were homozygous for the I allele and (0.47) were heterozygous (I/D) in Brazilian healthy children.^33^ Park et al. reported that genotype frequencies of *ACE1* D/D were 17.5%, I/D 45.0% and I/I 37.5% in Korean hypertensive adolescents.^22^ A recent meta-analysis performed by Aziz and Islam included 11 studies which investigated the distribution of *ACE* I/D rs1799752 alleles in different populations and evaluated the association between *ACE* I/D rs1799752 polymorphism and COVID-19 severity. Interestingly, the authors confirmed the racial variance in this polymorphism and concluded that high frequency of the *ACE* D-allele was associated with disease severity through different models.^39^

It is plausible to speculate that a functional *ACE1* I/D SNP could modulate the susceptibility and severity of COVID-19. Increased ACE levels in subjects carrying the D/D genotype would enhance the deleterious Angiotensin II/AT1 receptor-axis that is worsened by the down-regulation of the ACE2 receptors due to viral binding resulting in reduced generation of Angiotensin (1–7) and unopposed accumulation of Angiotensin II.^8^ This anomalous tuning of the systemic and local RAS signaling pathway has been reported to be involved in the pathogenesis of hypertension, renal failure, thrombosis, and severe ARDS.^40,41^ Moreover, the downregulated ACE2 expression results in an increased bradykinin promoting pulmonary vasoconstriction in SARS-CoV-2-induced ARDS via the bradykinin (B) 2 receptors. Some authors suggested that dysfunctional RAS and kallikrein–kinin system may aggravate COVID-19 progression, in particular during cytokine storm release.^42^

Experimental work demonstrated that ACE2 deficiency resulted in up-regulation of Angiotensin II-induced expression of pro-inflammatory cytokines such as interleukin (IL)-1β, IL-6, tumor necrosis factor -α, monocyte chemoattractant protein-1 and TGF-β that may contribute to the cytokine storm in humans; a major driver of illness severity during SARS-COV2 infection.^43,44^ However, an overly activated RAS may represent a part of complex multistep mechanism mediating abnormal inflammatory and thrombotic processes in COVID-19 to be further investigated.

In animal models, the use of ACE inhibitors or Angiotensin II receptor blockers was effective in decreasing acute lung injury and improving pulmonary function.^10^

Recent studies suggested that excessive soluble recombinant human ACE2 competitively bind the SARS-CoV-2 virus thus may block viral spreading and rescue the intra-cellular ACE2 activity. Whether or not combined with ACE-inhibitors, excessive soluble rhACE2 could be a potential therapeutic strategy to counteract unescapably unrestrained ACE activity to contrast SARS coronavirus-induced lung injury and ARDS.^30,45^

The small sample size of the study population was the first limitation in the current study. Second, the *ACE1* I/D SNP may represent linkage-disequilibrium with other polymorphisms in the *ACE1* gene directly affecting systemic ACE activity. Accordingly, we recommend adopting a genome-wide association study across various ethnic populations to investigate other SNPs with known functional significance and confirm the role of *ACE1* gene variation in COVID-19. Third, a serial measurement of serum ACE activity with concurrent evaluation of Angiotensin II and ACE2 levels might better correlate the RAS pathway with disease severity and outcome. Finally, lack of sufficient data about environmental risk factors for SARS-CoV-2 infection in a genetically susceptible child.

## Conclusion

The *ACE1* insertion/deletion polymorphism may confer susceptibility to SARS-CoV-2 infection in Egyptian children and adolescents. The *ACE1* Deletion allele and D/D genotype, being associated with higher systemic ACE activity may constitute independent risk factors for COVID-19 severity.

Finally, *ACE1* I/D genotyping may provide an opportunity for better risk stratification and help design further clinical trials reconsidering RAS-pathway antagonists in selected patients with COVID-19 to optimize intervention strategies and achieve more efficient targeted therapies.

## Supplementary Information

Table S1

Table S2

## Data Availability

All data generated during and/or analyzed during the current study are available from the corresponding author on reasonable request.
