# Supplementary material for: Association of ACE1 I/D polymorphism and susceptibility to COVID-19 in Egyptian children and adolescents
Source: Pediatr Res. 2024 Jan 4;96(5):1347–54. doi: 10.1038/s41390-023-02982-8 (PMC11521986; doi:10.1038/s41390-023-02982-8)
Supplement: Supplementary file 1 — Table S1 [file 41390_2023_2982_MOESM1_ESM.pdf]

Table S1: Comparison of clinical and laboratory variables between COVID-19 subgroups according to disease severity.

COVID-19 Severity	***Mild*** (n=147)	***Moderate*** (n=166)	***Severe & critical*** (n=147)	***P***
**Clinical manifestations, n (%)**				
Fever	147(100)	166 (100)	147(100)	NA
Tachycardia on admission	13 (9)	38 (23)	61 (41)	0.056
Tachypnea on admission	_	166 (100)	32 (21.7)	NA
Signs of LRTI	_	166 (100)	32 (21.7)	NA
Pneumonia	_	166 (100)	32 (21.7)	NA
Hypoxemia	_	_	69 (47)	NA
Dehydration	_	_	55 (37)	NA
Cyanosis	_	_	46 (31)	NA
Shock	_	_	32 (21.7)	NA
ARF	_	_	28 (19)	NA
Duration of fever (in days)	3 (2 - 5)	7 (5 - 9)	11 (7 - 21)	0.164
Duration of hospital stay	2 (1 - 6)	9 (7-15)	13 (10-21)	0.058
**Laboratory findings, median (IQR)**				
Procalcitonin (ng/ml),	9.8 (0.07-16.3)	12.8 (1.1-17.8)	16 (1.2-18.7)	0.146
D-Dimer (µg/mL)	0.21 (0.28–1.58)	0.74 (0.31– 2.2)	0.98 (0.36–3.5)	**0.02**
LDH (U/L)	203 (154 – 264)	207 (198 – 279)	245 (200 – 307)	0.372
CRP (mg/dl)	6.8 (0.76 – 21.8)	7.1 (0.88 – 23)	8 (0.94 – 25)	0.074
Serum Ferritin(ng/ml)	29 (17 –294)	38 (21 –356)	41 (26 –385)	0.343
WBCs, $\times 10^{9}$/L	7.2 (5.8-12.6)	7.3 (5.5-11.4)	6.5 (4.6-9.8)	0.287
Lymphocytes, $\times 10^{9}$/ L	2.6 (1.5-3.1)	1.4 (1.15-2.8)	1.1 (0.59-2.0)	**0.006**
Platelets, $\times 10^{9}$/ L	240 (194-340)	187 (165-320)	167 (141-312)	0.132
ALT (U/L)	16 (12-54)	19.6 (11-73)	21 (14-78)	0.085
AST (U/L)	21.6 (13-63)	25 (13.8 -89)	29 (14 -95)	0.176
Creatinine (µmol/L)	31.8 (22-49)	42 (29-58)	46.7 (32-78)	0.059

Abbreviations: NA; Non applicable, LRTI; Lower respiratory tract infection, IQR; interquartile range, LDH; Lactate dehydrogenase, WBC; White blood cell ;CRP; C-reactive protein, ALT; alanine aminotransferase, AST; aspartate aminotransferase.
Data are median (IQR) or n (%). *P* value < 0.05 indicates a significant difference.
