# Supplementary material for: Association of ACE1 I/D polymorphism and susceptibility to COVID-19 in Egyptian children and adolescents
Source: Pediatr Res. 2024 Jan 4;96(5):1347–54. doi: 10.1038/s41390-023-02982-8 (PMC11521986; doi:10.1038/s41390-023-02982-8)
Supplement: Supplementary file 2 — Table S2 [file 41390_2023_2982_MOESM2_ESM.pdf]

Table S2

Correlation between serum ACE levels and clinical-laboratory parameters in patients with COVID-19.

**Serum ACE** (pg/mL)	*R*	*P value*
age(years)	0.113	0.155
Female sex	0.068	0.712
Fever	0.043	0.589
Tachypnea	0.185	0.246
Tachycardia	0.196	0.148
Oxygen saturation	- 0.365	**0.02**
CRP, mg/dl	0.286	0.057
Proclcitonin, ng/ml	0.175	0.416
Serum Ferritin, ng/ml	0.091	0.135
Lactate dehydrogenase (LDH), U/L	0.085	0.384
D-Dimer , µg/Ml	0.276	0.055
White blood cell, $\times 10^{9}$/L	0.044	0.298
Lymphocytes, $\times 10^{9}$/L	- 0.374	**0.04**
Platelets, $\times 10^{9}$/L	0.094	0.725
ALT, U/L	0.116	0.432
AST, U/L	0.124	0.367
Creatinine, µmol/L	0.197	0.156

Abbreviations: CRP; C-reactive protein, ALT; alanine aminotransferase, AST; aspartate aminotransferase.
P value < 0.05 indicates a significant difference.
